# Comparison of Fecundity and Gall-Forming of the Horned-Gall Aphid, *Schlechtendalia chinensis* (Hemiptera: Aphididae) from Different Populations

**DOI:** 10.3390/insects16010100

**Published:** 2025-01-18

**Authors:** Xin Xu, Zhaohui Shi, Chang Tong, Shuxia Shao, Hongyuan Wei, Zixiang Yang

**Affiliations:** Yunnan Key Laboratory of Breeding and Utilization of Resource Insects, Key Laboratory of Breeding and Utilization of Resource Insects of National Forestry and Grassland Administration, Institute of Highland Forest Science, Chinese Academy of Forestry, Kunming 650224, China; xuxin1995614@163.com (X.X.); zhaohui1019847069@163.com (Z.S.); tongchang186@163.com (C.T.); shuxiashao@126.com (S.S.); 15953572385@163.com (H.W.)

**Keywords:** horned-gall aphid, Chinese gallnut, population, reproduction, gall-forming, biological characteristics

## Abstract

The horned-gall aphid, *Schlechtendalia chinensis*, is the most economically valuable Chinese gallnut aphid species, playing a decisive role in the production of Chinese gallnuts. The method of cultivating the gallnut species with artificial moss and increasing the yield of gallnuts after inoculation has been applied in the main producing areas of Chinese gallnuts. However, it is still unclear whether artificial cultivation affects the fecundity and gall-forming effect of *S. chinensis*. In this study, autumn migrant aphids of *S. chinensis* from wild, artificial and introduced populations were used as materials to cultivate and inoculate under the same environment. The number of male and female sexuales, fundatrices, the galls per tree, and the total weight of galls per tree in subsequent generations were analyzed, and differences in the fecundity and gall-forming effects of different populations were analyzed. The results showed that the fecundity of the wild population was stronger than that of the artificial population, and the number of aphids produced by a single spring migrant and the number of fundatrices increased by 75.86% and 81.62%, respectively. Compared with the introduced population, the survival rate of female sexuales in the local population was higher. Compared with the artificial population, the gall-forming effect of the wild population was better; the number of galls per tree, the weight of single gall, and the total weight of galls per tree increased by 68.33%, 50.77%, and 153.78%, respectively; and the gall preservation rate increased significantly. Artificial cultivation of *S. chinensis* will lead to a decrease in fecundity and gall-forming effect in subsequent generations, showing the degradation of the vitality of *S. chinensis*. Therefore, it is necessary to improve the effect of artificial cultivation of *S. chinensis* by adopting technical measures such as wild population or introduction.

## Simple Summary

The horned-gall aphid is an economically important insect and the primary species for Chinese gallnut production. Although artificial cultivation methods have been widely applied in major production areas, their impact on the fecundity and gall-forming ability of aphids remains unclear. This study compared wild, artificial and introduced populations of aphid under the same conditions and found that artificial cultivation leads to reduced fecundity and gall-forming ability in subsequent generations compared to wild populations. To improve the effectiveness of artificial cultivation, technical measures such as wild population or introduced are recommended.

## 1. Introduction

Insecta is the biggest group of animals on earth and is considered one of the biggest biological resources that has not been fully exploited by humans. The resource insect industry is a traditional Chinese advantageous industry, with significant ecological, economic, and social value [1]. The horned-gall aphid, *Schlechtendalia chinensis* (Hemiptera: Aphididae: Eriosomatinae), is an important resource insect that parasitizes its host, *Rhus chinensis*, forming galls known as Chinese gallnuts [2]. The main component of Chinese gallnuts is tannic acid, which is widely used in industries such as pharmaceuticals, chemicals, food, and electronics, making it a highly valuable economic resource [3,4]. However, the Chinese gallnut industry has long faced a shortage of raw materials due to severe labor shortages in production areas and the improvement of the production capacity of the deep-processing production line. Therefore, it has become an important challenge in production technology to increase the yield of gallnuts by artificially cultivating *S. chinensis* and inoculating them on *R. chinensis* to form galls.

*S. chinensis* has a complex life cycle, including various developmental stages such as spring migrant, sexual, fundatrix, fundatrigenia, autumn migrant, and overwintering nymph. Its lifecycle involves host switching between the summer host, Chinese sumac (*R. chinensis*), and the most suitable winter host, moss (*Plagiomnium maximoviczii*), and forming galls on *R. chinensis* with fundatrices [5,6]. Based on gall formation, the generation of *S. chinensis* can be categorized into in-gall generation and out-of-gall generation [7]. In the wild, winged spring migrants migrate from *P. maximoviczii* to the cracks of *R. chinensis* after overwintering to produce male and female sexuales. Mated female sexuales produce fundatrices approximately one month later, and the sexual stage does not require feeding. The newly born fundatrices quickly climb to the young shoots of *R. chinensis* to feed, inducing it to form closed galls. In the gall, fundatrices produce three generations of wingless fundatrigenia, whose increasing numbers cause the gall to enlarge. In autumn, the third generation of fundatrigenia develops into winged aphids, which migrate back to *P. maximoviczii* for overwintering, thereby restarting the next lifecycle [2,3]. Due to the significant impact of the natural environment on the growth and development of *S. chinensis* and the large differences in the habitats of *R. chinensis* and *P. maximoviczii*, the wild population faces considerable survival pressure. Based on the biological characteristics of *S. chinensis* and its interactions with its hosts, artificial cultivation technology has been developed to optimize moss cultivation for aphid overwintering, the collection and bagging of spring migrant aphids, and hanging inoculation bags, thus significantly increasing the aphid population.

However, during the artificial cultivation of *S. chinensis*, some traditional production areas have practiced long-term recycling of aphids. Whether this practice affects the fecundity and gall-forming effect of the offspring, thus affecting the yield of gallnut, is worthy of further exploration. In this study, the subsequent generations of *S. chinensis* from the wild population, artificial population, and introduced population were investigated under the same cultivation and inoculation conditions, and the number of sexuales and fundatrices, the galls per tree, and the total weight of galls per tree were analyzed. The fecundity and gall-forming effects of different populations of *S. chinensis* were evaluated, which provided basic data for the improvement of *S. chinensis* cultivation technology.

## 2. Materials and Methods

### 2.1. Insect Colonies and Host Plant

*S. chinensis* populations included the local wild and local artificial populations from Wufeng, Hubei Province, with three artificial introduced populations represented by the major production areas of Chinese gallnuts in the Wuling Mountain region: Sangzhi, Hunan Province; Youyang, Chongqing; and Shangyang, Shaanxi Province (Table 1). In September 2022, mature galls were collected from each production area, and autumn migrant aphids in the galls were transferred to *P. maximoviczii* for cultivation. During the overwintering period, the aphids were artificially cultivated using a soilless moss-based system. From March to April 2023, spring migrants emerging on the moss were collected and packed into aphid bags, with approximately 100 aphids per bag. These aphid bags were transported to Wufeng in Hubei Province and used for subsequent tests.

The experimental host tree was a three-year-old *R. chinensis* plantation, established in 2020 from the seeds of a single parent tree. It is located in Wufeng, Hubei Province (30°10′ N, 110°57′ E, altitude 1050 m). The trees were planted in a space of 1 m × 2 m and were uniformly managed during the three-year period.

### 2.2. Experimental Methods

#### 2.2.1. Storage and Hanging of Aphid Bags

The aphid bags were subject to two batches of hanging. The aphid bags from different populations were moistened and stored at room temperature and low temperature (approximately 4–8 °C lower than room temperature), respectively. When the fundatrices began to appear in the aphid bags, the bags were opened promptly and hung on the branch about 10 cm below the young shoots of *R. chinensis*, with one bag per tree. This allowed the fundatrix to crawl to a tender leaf, feed, and induce gall formation. The aphid bags from each population were hung continuously on five consecutive trees before rotating to bags from a different population. When the fundatrices began to appear in the aphid bags stored at low temperatures, the second batch of bags was hung following the same method, ensuring that the same tree received aphid bags from the same population by hanging twice.

#### 2.2.2. Statistics of the Number of *S. chinensis* in Aphid Bags

On the 10th day after spring migrants were bagged, 15 bags were randomly selected from each population, and the numbers of spring migrants, female sexuales, and male sexuales in each bag were counted and recorded. When fundatrices began to appear in the aphid bag, 15 bags were randomly selected from each population, and the number of fundatrices in the bag was counted.

#### 2.2.3. Investigation on the Number of Galls Induced by *S. chinensis*

The developmental stages of galls induced by *S. chinensis* in the in-gall generation were divided into three periods: early stage (June), middle stage (August), and late stage (October). Data statistics were recorded once in each period, including the number of galls per tree, galls per leaf, and compound leaves with galls per branch. This was repeated 30 times for each population. When the galls matured, all galls from individual trees of each population were collected to determine their total number and total weight. Additionally, the wall thickness of the gall was measured. This was repeated 10 times for each population.

### 2.3. Statistical Analysis

One-way analysis of variance (ANOVA) was used to compare the differences in the numbers of aphids and galls between different populations. Duncan’s new multiple range test (DMRT) was carried out to separate the means once there was significant difference among the datasets (α = 0.05). Linear correlation analysis was carried out between the gall preservation rate and the cultivation years of *S. chinensis*, the number of galls per tree and the number of fundatrices produced in-bag, the wall thickness of the gall and the latitude of the population’s location, and the number of galls and the weight of a single gall on single compound leaf. The female-to-male ratio of sexuales in different populations was tested for conformity to a 1:1 ratio using the Chi-square goodness of fit test. All statistical analyses were performed using IBM^®^ SPSS^®^ Statistics 21.0 software.

## 3. Results

### 3.1. Aphid Numbers in the Bag

**Figure 1:**
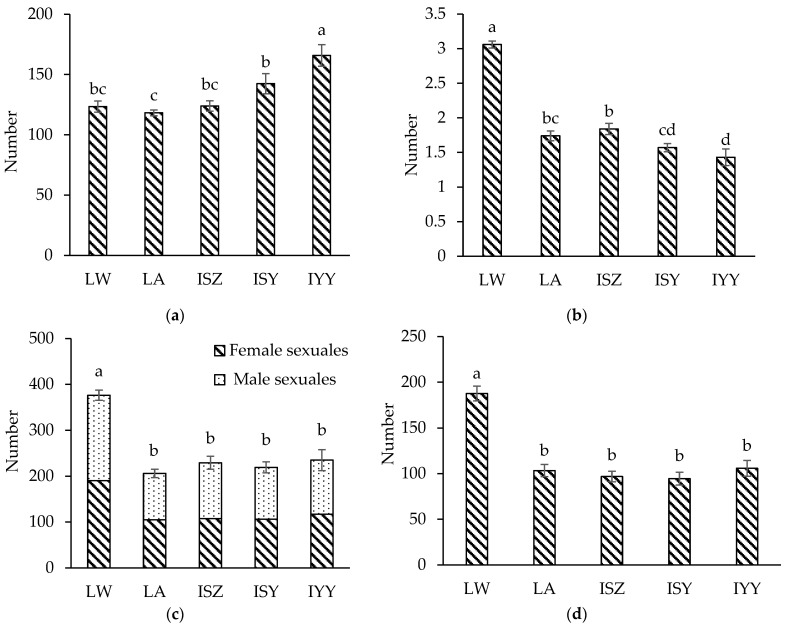
The number of spring migrant aphids (**a**), aphids produced by a single spring migrant (**b**), male and female sexuales (**c**), and fundatrices (**d**) in different populations of *Schlechtendalia chinensis*. Note: LW represents the local wild population; LA represents the local artificial population; ISZ represents the introduced population in Sangzhi; ISY represents the introduced population in Shanyang; IYY represents the introduced population in Youyang. Data in the figure are mean ± standard error. Those with different lowercase letters on the column indicate that there were significant differences in the numbers of different populations of the aphid (*p* < 0.05, DMRT method). The same applies below.

The average number of spring migrants in different populations of *S. chinensis* was more than 100 per bag, which met the requirements of the established Chinese gallnut industry standard (Figure 1a). The differences in the numbers of spring migrants in the bags of each population was due to artificial error in bagging and selecting them. There was no significant difference in the number of spring migrants between the local wild and artificial populations. Among all the local and introduced populations, the number of aphids produced by a single spring migrant in the local wild population was the highest (3.06 ± 0.05), which was 75.86% higher than that of the local artificial population (1.74 ± 0.07) (Figure 1b). According to the reproduction of each 100 spring migrants, the important reproductive indicators—the number of male sexuales, female sexuales, sexuales, and fundatrices in the bag—were significantly higher in the local wild population compared to the local artificial population, with increases of 75.93%, 73.78%, 74.84%, and 73.97%, respectively (Table 2). Moreover, the growth environment of the local wild population and artificial population was the same, which excluded the influence of external environment on the production of spring migrants, indicating that the fecundity of the wild population was stronger than that of the artificial population.

The proportion of male and female sexuales in all populations was consistent with 1:1 by the Chi-square goodness of fit test (χ^2^ = 0.05, 0.06, 0.87, 0.21, 0.00; *p* = 0.84, 0.78, 0.36, 0.64, 0.95). Among all the local and introduced populations, the total number of male and female sexuales in the local wild population was the highest (376.13 ± 11.08 per bag), which was 82.53% higher than that in the local artificial population (206.07 ± 9.15 per bag) (Figure 1c). The survival rates of female sexuales in both the local wild and artificial populations were similar and notably higher than those in the introduced population (Table 2). This difference may be attributed to the reduced survival rate of female aphids in the introduced population caused by the stress of long-distance transport in-bag. Since each female sexuale produces only one fundatrix, the number of fundatrices was highest in the local wild population (187.67 ± 8.10 per bag), which was 81.62% higher than that in the local artificial population (103.33 ± 6.69 per bag) (Figure 1d). There were no significant differences in the total number of sexuales and the number of fundatrices between the local and introduced artificial populations.

### 3.2. Gall Number Statistics

The number of galls in all local and introduced populations decreased significantly throughout the whole in-gall generation of *S. chinensis*. The average gall preservation rate was 45.89%, with the gall preservation rate from June to August being higher than that from August to October (Table 3). The main reason for the loss of galls may be the adverse external environment and the occurrence of pests and diseases during the intensive production process. In addition, population differences will also have a certain impact on it. Among all the local and introduced populations, the local wild population had the highest gall preservation rate, while the local artificial population had the lowest. The gall preservation rate was negatively correlated with the cultivation years of *S. chinensis* (r = −0.937, *p* = 0.019 < 0.05), that is, the more cultivation years, the lower the gall preservation rate.

**Figure 2:**
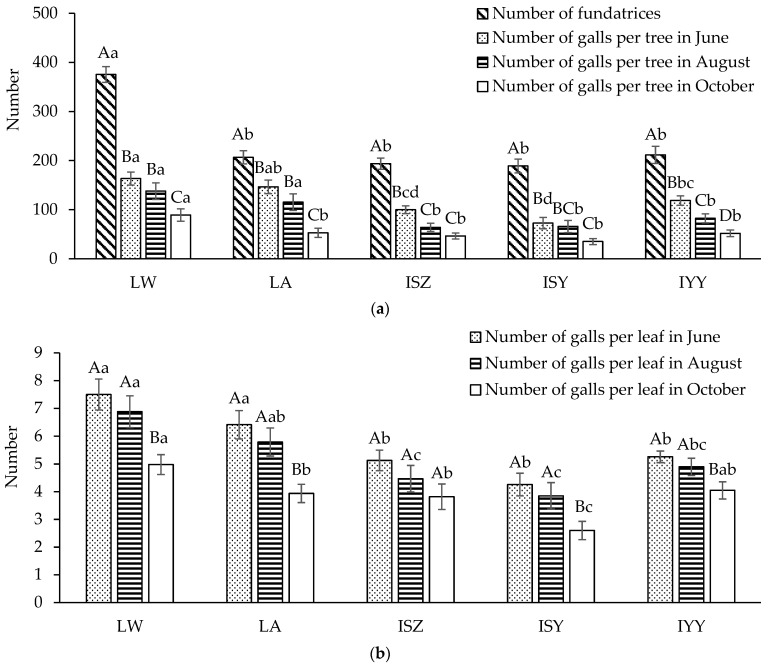

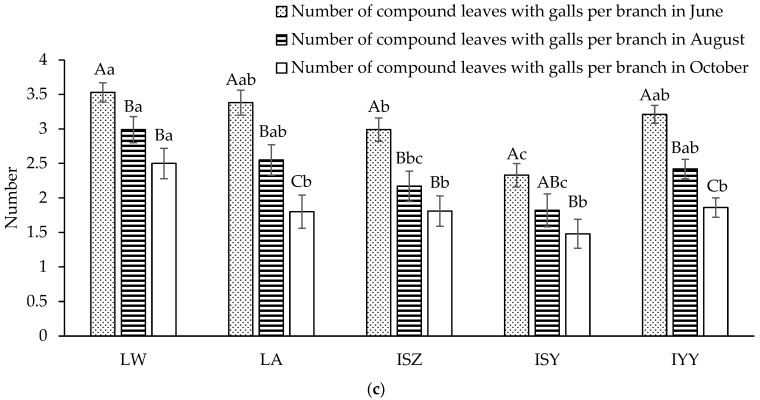
Comparison of the number of fundatrices per plant and the number of galls per plant (**a**), galls per leaf (**b**), and compound leaves with galls per branch (**c**) in different periods of different populations of *Schlechtendalia chinensis*. Note: LW represents the local wild population; LA represents the local artificial population; ISZ represents the introduced population in Sangzhi; ISY represents the introduced population in Shanyang; IYY represents the introduced population in Youyang. The same uppercase letter on the same group column indicates that the number of the same population in different periods is not significantly different at the 0.05 level (DMRT method). The same lowercase letters on the same type of column indicate that the number of different populations in the same period was not significantly different at the 0.05 level (DMRT method). The same applies below.

The October survey date coincided with the harvest period of commercial horned-galls, and thus the number of galls in October could be regarded as the yield of commercial horned-galls. During the early and middle stages of gall development, there was no significant difference in the number of galls per tree between the local wild and local artificial populations. However, in the late stage, the local wild population (89.28 ± 12.71) exhibited a significantly higher number of galls, with an increase of 68.33% compared to the local artificial population (53.04 ± 9.39) (Figure 2a). Moreover, the number of galls per tree in the local wild population was significantly higher than those in the introduced populations throughout the in-gall generation. Based on the gall-forming of each 100 spring migrants, the number of galls per tree in the local wild population in October was 61% higher than that in the local artificial population. The number of galls per tree was positively correlated with the number of fundatrices produced in-bag (r = 0.967, *p* = 0.007 < 0.05).

The number of fundatrices in the local wild population was 1.82 times that of the local artificial population, but the number of galls in the early stage was only 1.12 times that of the local artificial population. The gall-forming rate of fundatrices in the local wild population was only three-quarters of that in the local artificial population, which may be due to the limitation of the maximum environmental carrying capacity of the host plant, *R. chinensis*. Apart from the local wild population, the gall-forming rate of fundatrices in the local artificial population was significantly higher than that in the introduced population. The reason for the decrease in gall-forming ability in the introduced population may be due to the long-distance transport in-bag and phenological differences.

The number of galls per leaf and the number of compound leaves with galls per branch decreased significantly throughout the whole in-gall generation of *S. chinensis*. The preservation rate of galls per leaf was 68.07%, and the preservation rate of compound leaves with galls per branch was 61.21%. During the early and middle stages of gall development, there was no significant difference in the number of galls per leaf and the number of compound leaves with galls per branch between the local wild and the local artificial populations. However, in the late stage of gall development, the local wild population showed significantly higher numbers than the local artificial population, with increases of 26.40% and 38.89%, respectively (Figure 2b,c). Moreover, the local wild population exhibited significantly higher numbers of galls per leaf and compound leaves with galls per branch compared to the introduced populations throughout the in-gall generation. During the in-gall generation, the local wild population was superior to the local artificial population and the introduced populations in the number and the preservation rate of galls per tree, galls per leaf, and compound leaves with galls per branch, indicating that the gall-forming effect of the local wild population was significantly better than those of the local artificial population and the introduced population.

The wall thickness and weight of galls varied significantly among different populations, which may be related to the geographical environment of the populations (Table 4). In the four artificial populations, gall wall thickness showed a negative correlation with the latitude of the population’s location (r = −0.741, *p* = 0.259). There was no significant correlation between the number of galls and the weight of a single gall on single compound leaf (r = −0.083, *p* = 0.894). The total gall weight per tree of the local wild population was 2.54 times that of the local artificial population. Based on the gall-forming of each 100 spring migrants, the total gall weight per tree of the local wild population was 2.43 times that of the local artificial population. The local wild population was significantly better than the local artificial population in gall wall thickness, single gall weight, the number of galls per tree, and the total gall weight per tree.

## 4. Discussion

In the long-term artificial cultivation process, the fecundity and gall-forming of the artificial population of *S. chinensis* decreased significantly compared with the wild population, which confirmed the view that the vitality of the aphids will be degraded after prolonged use. The decrease in the fecundity and gall-forming of the artificial population is mainly manifested in the reduction of the number of aphids produced by spring migrants and the gall preservation rate. After 13 years of continuous use, the number of aphids produced by spring migrants and the gall preservation rate in the local artificial population decreased by 42.80% and 33.71%, respectively, compared with the local wild population. The differences in populations may be related to genetic or environmental factors and their interactions [8,9,10]. In terms of genetics, the reproductive mode of the aphid is cyclic parthenogenesis, with bisexual reproduction occurring only in the sexual stage. During artificial cultivation, the sexual stage takes place within the aphid bag, increasing the likelihood of inbreeding. This leads to a decrease in the genetic diversity of the artificial population, manifested as declines in viability, fecundity, stress resistance, and other characteristics. In terms of environments, the aphid has a complex heteroecious and holocyclic life cycle, which requires switching between the summer host and the winter host. The wild population, through rigorous natural selection, typically retains individuals with more competitiveness in survival and reproductive efficiency. In contrast, the artificial population in controlled environments lacks natural selective pressures, which may lead to the accumulation of adverse traits, resulting in declines in reproductive and gall-forming efficiency. In other commercially cultured insect populations, the biological characteristics of artificial populations are also degraded to varying degrees compared to wild populations. The fecundity, parasitism efficiency, longevity, and host suitability indices of the artificial population of *Cotesia flavipes* were lower than those of the wild population [11,12,13]. The mating frequency as well as the length, width, and weight of the pupae of the artificial population of *Anastrepha obliqua* were significantly lower than those of the wild population. Furthermore, the sexual competitiveness of the artificial population was also inferior to that of the wild population [14,15,16]. Other species of parasitoids have also been shown to exhibit greater reproductive capacity in the wild than in the laboratory [17,18,19]. However, some studies have found that artificial populations of *Anastrepha striata* demonstrate greater reproductive capacity during the oviposition period compared to wild populations [20].

Based on the results of this study, improving key technical measures in the artificial cultivation process of *S. chinensis* can significantly enhance its utilization efficiency, thereby increasing the yield of Chinese gallnuts. First of all, using the wild population for cultivating is the most economical and effective approach. The wild population generally exhibits stronger reproductive and gall-forming abilities, which can significantly increase the number of sexuales and the gall preservation rate, directly promoting the increase of gallnut yield. Secondly, optimizing the time interval between the two batches of aphid bag hanging is critical. By extending the time interval between the two aphid bags to an appropriate length, the number of compound leaves with galls per branch could reach seven. This will not only help to effectively disperse the galling sites of fundatrices and avoid excessive concentration of resources, but also maximize the carrying capacity of the *R. chinensis* compound leaves for *S. chinensis*, and improve the utilization efficiency of the aphid. Finally, the local population should be used as much as possible in the cultivating process, which can reduce the negative effects of long-distance transportation and phenological differences in the survival ability of female sexuales and the gall-forming ability of fundatrices. Scientific and rational selection of high-quality populations and optimization of cultivation management and timing are not only helpful to improve the utilization efficiency of aphids, but also to greatly increase the yield of gallnuts, thereby improving the economic benefits of the Chinese gallnut industry and providing strong support for development toward high and stable production.

## 5. Conclusions

In summary, long-term artificial cultivation will lead to the degradation of the biological characteristics of insect populations, manifested as decreased fecundity, adaptability, and viability. The artificial population of *S. chinensis* exhibited significantly lower reproductive and gall-forming abilities compared to the wild population. This is particularly evident in the decrease in aphid numbers produced by spring migrants (42.80% lower than the wild population) and gall preservation rate (33.71% lower than the wild population). Such degradation may be related to the decrease in genetic diversity (e.g., inbreeding) and the lack of natural selection pressure. This study evaluated the effects of wild, artificial, and introduced populations on the biological characteristics of *S. chinensis* and provided a scientific basis for improving Chinese gallnut cultivation technology.

## Figures and Tables

**Table 1:** Basic information on different populations of *Schlechtendalia chinensis*.

Population	Location	The Latitude and Longitude of the Population		Years of Cultivation	Date of Aphid Collection	Date of the First Hanging Bag	Date of the Second Hanging Bag
		Longitude/°	Latitude/°				
The local wild population	Wufeng, Hubei Province	111.05	30.17	0	31 March	5 May	11 May
The local artificial population	Wufeng, Hubei Province			13	27 March	29 April	2 May
The introduced population in Sangzhi	Sangzhi, Hunan Province	110.17	29.41	2	27 March	26 April	2 May
The introduced population in Shanyang	Shangyang, Shaanxi Province	109.89	33.54	4	9 April	8 May	11 May
The introduced population in Youyang	Youyang, Chongqing	108.77	28.85	5	27 March	26 April	2 May

**Table 2:** Fecundity in different populations of *Schlechtendalia chinensis*.

Population	Per 100 Spring Migrant Aphids					
	Number of Male Sexuales	Number of Female Sexuales	Number of Sexuales	Survival Rate of Female Sexuales/%	Number of Fundatrices	Gall-Forming Rate of Fundatrices/%
The local wild population	150.64	153.99	304.63	98.70	152.00	43.56
The local artificial population	85.63	88.61	174.24	98.60	87.37	70.89
The introduced population in Sangzhi	98.18	86.77	184.94	90.08	78.17	51.56
The introduced population in Shanyang	79.30	74.58	153.88	89.01	66.38	31.33
The introduced population in Youyang	71.05	70.65	141.69	90.32	63.81	56.08

**Table 3:** The gall-forming effect in different populations of *Schlechtendalia chinensis*.

Population	Per 100 Spring Migrant Aphids						
	Number of Galls per Tree in June	Number of Galls per Tree in August	Number of Galls per Tree in October	Gall Preservation Rate from June to August/%	Gall Preservation Rate from August to October/%	Gall Preservation Rate/%	The Total Weight of Galls per Tree/g
The local wild population	66.21	55.81	36.15	84.29	64.77	54.61	390.78
The local artificial population	61.93	48.80	22.42	78.79	45.94	36.20	160.75
The introduced population in Sangzhi	40.31	25.85	18.74	64.14	72.50	46.51	151.98
The introduced population in Shanyang	25.54	23.15	12.37	90.64	53.43	48.44	161.92
The introduced population in Youyang	35.79	24.87	15.63	69.49	62.85	43.68	180.37

**Table 4:** Quality of galls in different populations of *Schlechtendalia chinensis*.

Population	Wall Thickness/mm	Single Gall Weight/g	Number of Galls per Tree	The Total Weight of Galls per Tree/g
The local wild population	1.96 ± 0.03 b	10.81 ± 0.66 a	89.28 ± 12.71 a	965.12
The local artificial population	1.73 ± 0.03 c	7.17 ± 0.75 b	53.04 ± 9.39 b	380.30
The introduced population in Sangzhi	1.74 ± 0.02 c	8.11 ± 0.65 b	46.46 ± 6.19 b	376.79
The introduced population in Shanyang	1.60 ± 0.05 d	13.09 ± 1.23 a	35.23 ± 6.11 b	461.16
The introduced population in Youyang	2.12 ± 0.05 a	11.54 ± 0.98 a	51.83 ± 6.71 b	598.12

Data in the table are mean ± standard error. The same column data with different lowercase letters show significant difference (*p* < 0.05, DMRT method).

## Data Availability

Data are contained within the article.
